# The complete mitochondrial genome of *Neolissochilus benasi* (Cypriniformes: Cyprinidae)

**DOI:** 10.1080/23802359.2019.1703566

**Published:** 2020-01-10

**Authors:** Wei Gu, Gefeng Xu, Tianqing Huang, Bingqian Wang

**Affiliations:** a Heilongjiang River Fisheries Research Institute, Chinese Academy of Fishery Sciences, Harbin, PR China;; b Dianze Aquaculture Company of Huize, Huize, PR China

**Keywords:** *Neolissochilus benasi*, mitochondrial genome, phylogeny

## Abstract

In this study, the complete mitochondrial genome of *Neolissochilus benasi* has been determined by polymerase chain reaction method for the first time. The overall base composition of *N. benasi* mitogenome is 31.8% for A, 27.4% for C, 15.9% for G and 25.0% for T. The percentage of G + C content is 41.3%. The mitogenome is a circular DNA molecule of 16 583 bp in length with a D-loop region and contains 22 transfer RNA (tRNA) genes, two ribosomal RNA (rRNA) genes and 13 protein-coding genes. The mitochondrial genome sequencing for *N. benasi* in this study provides important molecular data for further evolutionary analysis for Cyprinoidea.

*Neolissochilus benasi*, which belongs to order Cyprinoidea, family Cyprinidae, genus *Neolissochilus*, is a new cyprinid species which was discovered from a spring at Amojiang River in Jingdong County, Yunnan, China (Chu and Chen 1989). This species is endemic to the Karst area of the Yunnan-Guizhou Plateau.

*Neolissochilus benasi* samples of this study were collected from Amojiang River in Jingdong county of Yunnan Province in China (23°33′ N; 101°24′ E). The specimen is stored in Fin strip library, Chinese Academy of Fishery Sciences and its accession number is CAFS2019062. The genome DNA was extracted following the traditional phenol-chloroform method (Taggart et al. 1992). Twenty-six primers were designed to amplify the PCR products for sequencing. The sequencing results were then assembled using ContigExpress 9.0 software (New York, NY, USA). The transfer RNA (tRNA) genes were identified using the program tRNAscan-SE 1.21 (http://lowelab.ucsc.edu/tRNAscan-SE.). The locations of protein-coding genes were determined by comparing with the corresponding known sequences of other Neolissochilus fish species.

The complete mitochondrial genome length of *N. benasi* was 16 583 bp in length (GenBank accession number MN598560). It consisted of 13 protein-coding genes, 2 rRNA genes, 22 tRNA genes and one D-loop region. The overall base composition of the mitogenome is 31.8% for A, 27.4% for C, 15.9% for G and 25.0% for T. The percentage of G + C content is 41.3%. To validate the phylogenetic position of *N. benasi*, we perform multiple sequence alignment and MEGA 6.0 (Tamura et al. 2013) to construct a neighbor-joining tree containing complete mitochondrial genome DNA of 20 species in Cyprinoidea. As shown in the phylogenetic tree (Figure 1), our sequence was clustered in genus *Neolissochilus*, including *N. hexagonolepis* (KU380329.1), *N. soroides* (AP011314.1) and *N. stracheyi* (NC_031555.1).

**Figure 1.**
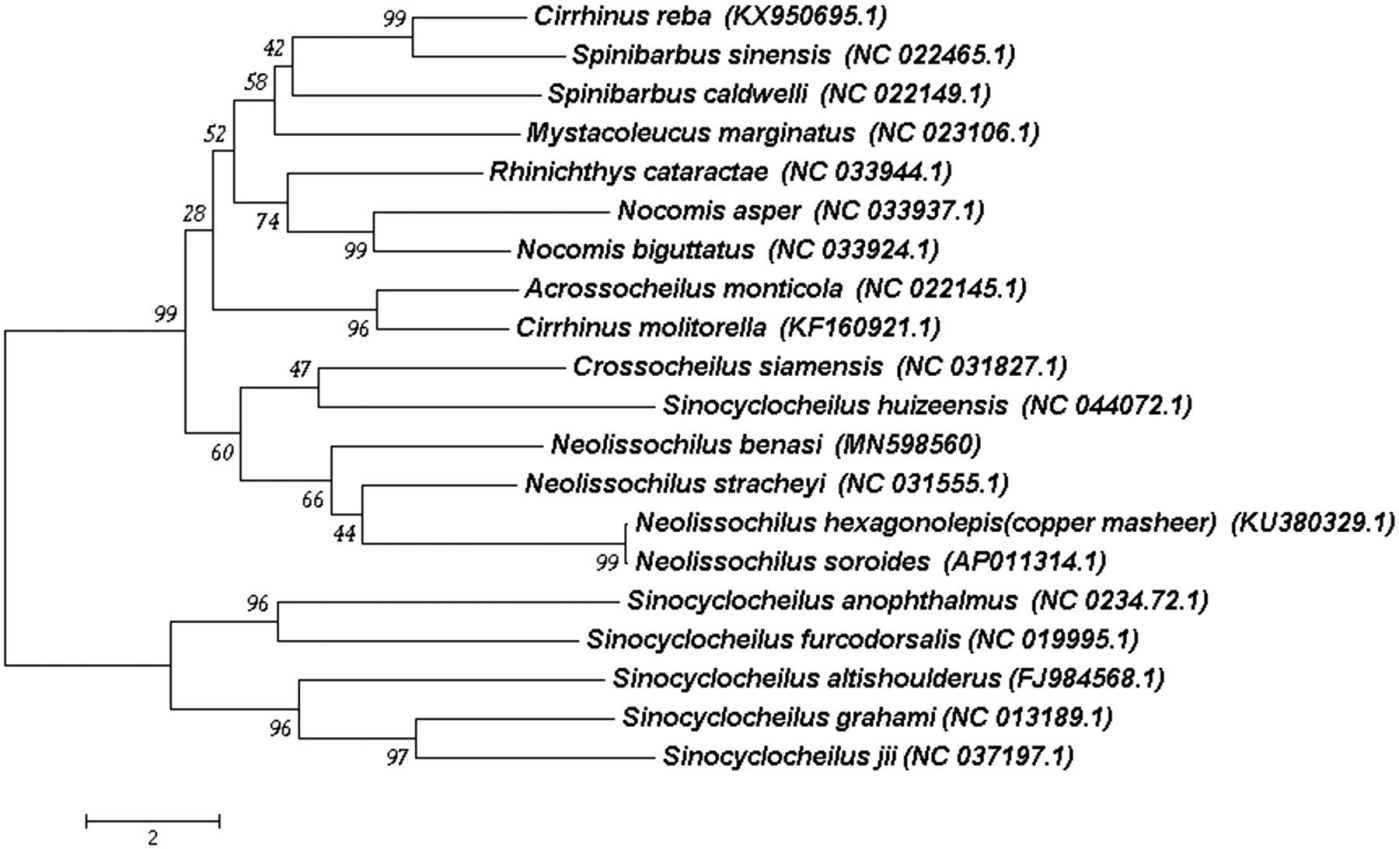
A neighbor-joining (NJ) tree of 20 species form Cyprinodea was constructed based on complete mitochondrial genome data.The analyzed species and corresponding NCBI accession numbers are as follows: *Cirrhinus reba* (KX950695.1), *Spinibarbus sinensis* (NC_022465.1), *Spinibarbus caldwelli* (NC_022149.1), *Mystacoleucus marginatus* (NC_023106.1), *Rhinichthys cataractae* (NC_033944.1), *Nocomis asper* (NC_033937.1), *Nocomis biguttatus* (NC_033924.1), *Acrossocheilus monticola* (NC_022145.1), *Cirrhinus molitorella* (KF160921.1), *Crossocheilus siamensis* (NC_031827.1), *Sinocyclocheilus huizeensis* (NC_044072.1), *Neolissichilus benasi* (MN598560), *Neolissichilus stracheyi* (NC_031555.1), *Neolissichilus hexagonolepis* (copper masheer) (KU380329.1), *Neolissichilus soroides* (AP011314.1), *Sinocyclocheilus anophthalmus* (NC_0234.72.1), *Sinocyclocheilus furcodorsalis* (NC_019995.1), *Sinocyclocheilus altishoulderus* (FJ984568.1), *Sinocyclocheilus grahami* (NC_013189.1), *Sinocyclocheilus jii* (NC_037197.1).
